# (*E*)-6-Meth­oxy-9-methyl-1,2,3,4-tetra­hydro-9*H*-carbazol-4-one oxime

**DOI:** 10.1107/S1600536808020047

**Published:** 2008-07-05

**Authors:** Wei Sheng, Qi-Hong Zhang, Zhui-Bai Qiu

**Affiliations:** a Department of Medicinal Chemistry, School of Pharmacy, Fudan University, 138 Yixueyuan Road, Shanghai 200032, People’s Republic of China

## Abstract

The title compound, C_14_H_16_N_2_O_2_, is dimerized by inversion-related inter­molecular O—H⋯O and O—H⋯N hydrogen bonding. There is also an intra­molecular C—H⋯N bond, resulting in a six-membered ring.

## Related literature

For general background, see: Hester (1967 ▶, 1970 ▶). For related literature, see: Sheng *et al.* (2008 ▶).
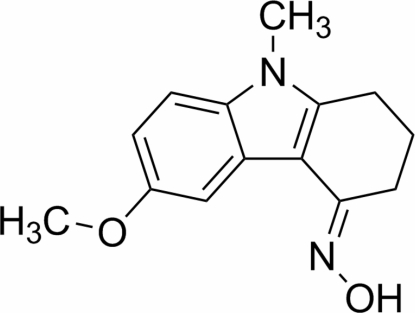


## Experimental

### 

#### Crystal data

C_14_H_16_N_2_O_2_
*M* *_r_* = 244.29
Monoclinic, 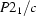

*a* = 8.833 (5) Å
*b* = 6.460 (4) Å
*c* = 22.247 (12) Å
β = 104.14 (2)°
*V* = 1231.0 (12) Å^3^
*Z* = 4
Mo *K*α radiation
μ = 0.09 mm^−1^
*T* = 293 (2) K
0.15 × 0.08 × 0.08 mm

#### Data collection

Bruker SMART APEX CCD area-detector diffractometer
Absorption correction: multi-scan (*SADABS*; Sheldrick, 1996 ▶) *T*_min_ = 0.987, *T*_max_ = 0.993
5647 measured reflections
2626 independent reflections
1396 reflections with *I* > 2σ(*I*)
*R*_int_ = 0.037

#### Refinement

*R*[*F* ^2^ > 2σ(*F* ^2^)] = 0.055
*wR*(*F* ^2^) = 0.165
*S* = 0.89
2626 reflections
169 parameters
2 restraints
H atoms treated by a mixture of independent and constrained refinement
Δρ_max_ = 0.61 e Å^−3^
Δρ_min_ = −0.32 e Å^−3^

### 

Data collection: *SMART* (Bruker, 2000 ▶); cell refinement: *SMART*; data reduction: *SAINT* (Bruker, 2000 ▶); program(s) used to solve structure: *SHELXTL* (Sheldrick, 2008 ▶); program(s) used to refine structure: *SHELXTL*; molecular graphics: *SHELXTL* and *ORTEP-3* (Farrugia, 1997 ▶); software used to prepare material for publication: *SHELXTL*.

## Supplementary Material

Crystal structure: contains datablocks global, I. DOI: 10.1107/S1600536808020047/om2245sup1.cif

Structure factors: contains datablocks I. DOI: 10.1107/S1600536808020047/om2245Isup2.hkl

Additional supplementary materials:  crystallographic information; 3D view; checkCIF report

## Figures and Tables

**Table 1:** Hydrogen-bond geometry (Å, °)

*D*—H⋯*A*	*D*—H	H⋯*A*	*D*⋯*A*	*D*—H⋯*A*
C5—H5⋯N1	0.93	2.76	3.236 (3)	113
O1—H1*X*⋯O1^i^	0.827 (18)	2.54 (3)	3.177 (5)	134 (3)
O1—H1*X*⋯N1^i^	0.827 (18)	2.00 (2)	2.810 (4)	166 (4)

Symmetry code: (i) 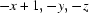.

**Fig. 1.**
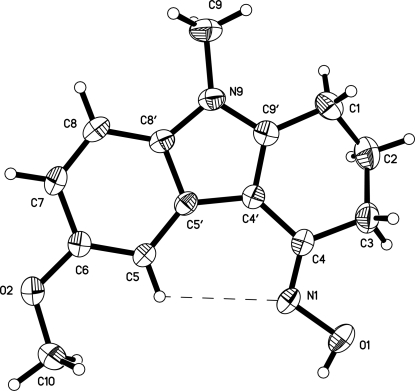
The molecular structure of the title compound, showing ellipsoids at the 20% probability level.

**Fig. 2.**
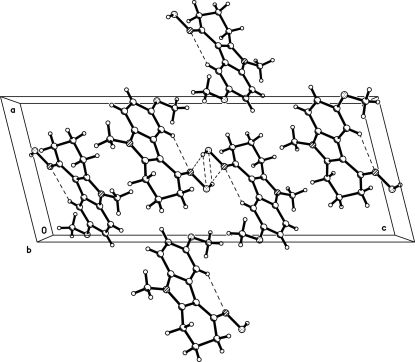
A view of the crystal packing, showing the hydrogen-bonding network.

## supplementary crystallographic information

### Comment

The most famous of the rearrangements in which *R* migrates from carbon to nitrogen is undoubtedly the conversion of ketoximes to N-subsituted amides, the Beckmann rearrangement. The most interesting feature of the rearrangement is that, it is not the nature (*e.g.* relative electron-releasing ability)but the stereochemical arrangment of the *R* and *R*'groups that determines which of them migrates. Almost without exception it is found to be the *R* group *anti* to the OH group that migrates from C to N. Thus, the structure of the amide produced is quite often used to establish the configuration of the oxime from which it was derived.

Surprisingly, in our study we obtained two different amides from one oxime by applying two different Beckmann conditions. This particular oxime, 6-methoxy -9-methyl-1,2,3,9-tetrahydro-4*H*-carbazol-4-one oxime (I), is found to yield only 6-methyl-9-methoxy-2,3,4,5-tetrahydroazepino[4,3-*b*]indol- 1(6*H*)-one (II) while treated with polyphophoric acids. However, by converting the OH group of (I) into a better leaving tosyl group followed a catalysis using Al_2_O_3_, (I) undergoes the rearrangement to 6-methyl-9- methoxy-3,4,5,6-tetrahydroazepino[3,2-*b*]indol-2(1*H*)-one (III) exclusively. Here we report the crystal structure of (I) in order to get a better understanding of the mechanism of this peculiar process.

Fig.1. shows the molecular structure of the title compound (I), which is almost planar and has the (E)-configuration. As shown in Fig. 2, the title compound is dimerized by inversion of (*E*)-6-methoxy-9-methyl-1,2,3,9-tetrahydro-4*H*-carbazol-4-one oxime through intermolecular H-bond *viz* O1—H1X···O1^i^ and O1—H1X···N1^i^[symmetry code i = -*x* + 1,-*y*,-*z*]. There is also an intramolecular hydrogen bond of C5—H5···N1 resulting in a six-membered ring.

### Experimental

The title compound (I) was prepared in three steps as follows. Firstly, with use of a method described by Sheng *et al.* (2008), 6-Methoxy-1,2,3,9- tetrahydro-4*H*-carbazol-4-one (IV) was prepared as starting material. Then,(IV) was methylated using dimethyl sulfate in a mixed solution of acetone and NaOH aq. to give 6-methoxy-9-methyl-1,2,3,9-tetrahydro-4*H*-carbazol-4-one (V). A mixture of (V) and hydroxylamine hydrochloride was dissolved in methanol and the solution was refluxed with a catalytic amount of pyridine. The crude product of the title compound was recrystallized in acetone to afford colorless prismatic crystals suitable for X-ray analysis.

### Refinement

The H atoms bonded to N and O in the carbazolone oxime were located in a difference map and refined with distance restraints of O—H = 0.82 (2) and N—H = 0.89 (2) Å. The H atoms attached to O and all carbon-bound H atoms were placed in calculated positions and refined as riding; O—H=0.82 and C—H=0.93–0.98 Å;*U*_iso_(H) = *xU*_eq_(parent atom) where *x*=1.5 for O and 1.2 for C.

### Crystal data

**Table:**

C_14_H_16_N_2_O_2_	*F*_000_ = 520
*M**_r_* = 244.29	*D*_x_ = 1.318 Mg m^−^^3^
Monoclinic, *P*2_1_/*c*	Mo *K*α radiation λ = 0.71073 Å
Hall symbol: -P 2ybc	Cell parameters from 777 reflections
*a* = 8.833 (5) Å	θ = 2.4–22.7º
*b* = 6.460 (4) Å	µ = 0.09 mm^−^^1^
*c* = 22.247 (12) Å	*T* = 293 (2) K
β = 104.14 (2)º	Prism, colorless
*V* = 1231.0 (12) Å^3^	0.15 × 0.08 × 0.08 mm
*Z* = 4	

### Data collection

**Table:**

Bruker SMART APEX CCD area-detector diffractometer	2626 independent reflections
Radiation source: fine-focus sealed tube	1396 reflections with *I* > 2σ(*I*)
Monochromator: graphite	*R*_int_ = 0.037
*T* = 293(2) K	θ_max_ = 27.0º
φ and ω scans	θ_min_ = 1.9º
Absorption correction: multi-scan(SADABS; Sheldrick, 1996)	*h* = −11→11
*T*_min_ = 0.987, *T*_max_ = 0.993	*k* = −8→8
5647 measured reflections	*l* = −27→17

### Refinement

**Table:**

Refinement on *F*^2^	Secondary atom site location: difference Fourier map
Least-squares matrix: full	Hydrogen site location: inferred from neighbouring sites
*R*[*F*^2^ > 2σ(*F*^2^)] = 0.055	H atoms treated by a mixture of independent and constrained refinement
*wR*(*F*^2^) = 0.165	*w* = 1/[σ^2^(*F*_o_^2^) + (0.0985*P*)^2^] where *P* = (*F*_o_^2^ + 2*F*_c_^2^)/3
*S* = 0.89	(Δ/σ)_max_ < 0.001
2626 reflections	Δρ_max_ = 0.61 e Å^−^^3^
169 parameters	Δρ_min_ = −0.32 e Å^−^^3^
2 restraints	Extinction correction: none
Primary atom site location: structure-invariant direct methods	

### Special details

**Table:**

Geometry. All e.s.d.'s (except the e.s.d. in the dihedral angle between two l.s. planes) are estimated using the full covariance matrix. The cell e.s.d.'s are taken into account individually in the estimation of e.s.d.'s in distances, angles and torsion angles; correlations between e.s.d.'s in cell parameters are only used when they are defined by crystal symmetry. An approximate (isotropic) treatment of cell e.s.d.'s is used for estimating e.s.d.'s involving l.s. planes.
Refinement. Refinement of *F*^2^ against ALL reflections. The weighted *R*-factor *wR* and goodness of fit *S* are based on *F*^2^, conventional *R*-factors *R* are based on *F*, with *F* set to zero for negative *F*^2^. The threshold expression of *F*^2^ > σ(*F*^2^) is used only for calculating *R*-factors(gt) *etc*. and is not relevant to the choice of reflections for refinement. *R*-factors based on *F*^2^ are statistically about twice as large as those based on *F*, and *R*- factors based on ALL data will be even larger.

### Fractional atomic coordinates and isotropic or equivalent isotropic displacement parameters (Å^2^)

**Table:**

	*x*	*y*	*z*	*U*_iso_*/*U*_eq_	
O1	0.3608 (3)	−0.1623 (4)	−0.01619 (11)	0.0908 (7)	
H1X	0.392 (4)	−0.054 (4)	0.0026 (18)	0.127 (16)*	
O2	1.0030 (2)	0.1510 (3)	−0.09437 (10)	0.0781 (6)	
N1	0.4790 (2)	−0.1762 (4)	−0.04926 (10)	0.0618 (6)	
C1	0.4333 (3)	−0.7039 (4)	−0.16677 (13)	0.0631 (7)	
H1A	0.4711	−0.8230	−0.1409	0.076*	
H1B	0.4036	−0.7496	−0.2096	0.076*	
C2	0.2945 (4)	−0.6123 (6)	−0.14878 (16)	0.0868 (10)	
H2A	0.2228	−0.7231	−0.1455	0.104*	
H2B	0.2410	−0.5203	−0.1816	0.104*	
C3	0.3335 (3)	−0.4929 (5)	−0.08831 (13)	0.0656 (7)	
H3A	0.2402	−0.4226	−0.0834	0.079*	
H3B	0.3647	−0.5901	−0.0543	0.079*	
C4	0.4606 (2)	−0.3367 (4)	−0.08413 (11)	0.0485 (6)	
C4'	0.5725 (2)	−0.3745 (3)	−0.12087 (9)	0.0422 (5)	
C5'	0.7093 (2)	−0.2625 (3)	−0.12699 (9)	0.0407 (5)	
C5	0.7843 (2)	−0.0819 (4)	−0.10084 (10)	0.0445 (5)	
H5	0.7437	−0.0036	−0.0734	0.053*	
C6	0.9189 (3)	−0.0218 (4)	−0.11637 (11)	0.0511 (6)	
C7	0.9790 (3)	−0.1381 (4)	−0.15859 (12)	0.0587 (7)	
H7	1.0704	−0.0947	−0.1685	0.070*	
C8	0.9066 (3)	−0.3127 (4)	−0.18535 (11)	0.0536 (6)	
H8	0.9468	−0.3879	−0.2136	0.064*	
C8'	0.7708 (2)	−0.3758 (4)	−0.16943 (9)	0.0441 (5)	
C9	0.7019 (3)	−0.6996 (4)	−0.23323 (12)	0.0674 (8)	
H9A	0.6501	−0.8262	−0.2277	0.101*	
H9B	0.8117	−0.7251	−0.2273	0.101*	
H9C	0.6599	−0.6479	−0.2744	0.101*	
N9	0.6779 (2)	−0.5471 (3)	−0.18818 (8)	0.0499 (5)	
C9'	0.5588 (3)	−0.5450 (4)	−0.15895 (10)	0.0477 (6)	
C10	0.9545 (3)	0.2735 (4)	−0.04983 (13)	0.0669 (7)	
H10A	0.8514	0.3259	−0.0673	0.100*	
H10B	1.0254	0.3872	−0.0379	0.100*	
H10C	0.9536	0.1907	−0.0141	0.100*	

### Atomic displacement parameters (Å^2^)

**Table:**

	*U*^11^	*U*^22^	*U*^33^	*U*^12^	*U*^13^	*U*^23^
O1	0.0865 (15)	0.1012 (18)	0.1098 (17)	−0.0189 (14)	0.0724 (14)	−0.0310 (15)
O2	0.0703 (12)	0.0740 (13)	0.1042 (15)	−0.0285 (10)	0.0485 (11)	−0.0276 (11)
N1	0.0588 (13)	0.0686 (15)	0.0708 (14)	−0.0054 (11)	0.0408 (11)	−0.0104 (12)
C1	0.0635 (15)	0.0564 (16)	0.0647 (16)	−0.0101 (13)	0.0067 (13)	−0.0052 (13)
C2	0.0733 (18)	0.091 (2)	0.101 (2)	−0.0302 (17)	0.0300 (17)	−0.0095 (18)
C3	0.0569 (15)	0.0730 (19)	0.0708 (17)	−0.0128 (14)	0.0233 (13)	0.0026 (14)
C4	0.0447 (12)	0.0537 (15)	0.0499 (13)	0.0003 (11)	0.0167 (10)	0.0059 (12)
C4'	0.0406 (11)	0.0448 (13)	0.0413 (12)	0.0003 (10)	0.0104 (9)	0.0019 (10)
C5'	0.0384 (11)	0.0489 (13)	0.0354 (11)	0.0046 (10)	0.0100 (9)	0.0029 (10)
C5	0.0441 (12)	0.0484 (14)	0.0448 (12)	−0.0004 (10)	0.0185 (10)	−0.0026 (10)
C6	0.0472 (12)	0.0525 (15)	0.0574 (14)	−0.0061 (11)	0.0198 (11)	−0.0045 (12)
C7	0.0480 (13)	0.0679 (18)	0.0688 (16)	−0.0044 (13)	0.0311 (12)	−0.0021 (14)
C8	0.0530 (13)	0.0642 (17)	0.0496 (13)	0.0078 (12)	0.0243 (11)	−0.0033 (12)
C8'	0.0434 (12)	0.0499 (14)	0.0391 (11)	0.0055 (10)	0.0102 (9)	−0.0019 (10)
C9	0.0851 (19)	0.0615 (17)	0.0577 (15)	0.0048 (14)	0.0216 (14)	−0.0169 (13)
N9	0.0531 (11)	0.0521 (12)	0.0450 (11)	0.0026 (10)	0.0132 (9)	−0.0093 (9)
C9'	0.0473 (12)	0.0495 (14)	0.0444 (12)	0.0018 (11)	0.0074 (10)	0.0019 (11)
C10	0.0744 (17)	0.0602 (17)	0.0676 (17)	−0.0170 (14)	0.0204 (14)	−0.0140 (14)

### Geometric parameters (Å, °)

**Table:**

O1—N1	1.419 (3)	C5'—C5	1.397 (3)
O1—H1X	0.827 (18)	C5'—C8'	1.405 (3)
O2—C6	1.364 (3)	C5—C6	1.373 (3)
O2—C10	1.414 (3)	C5—H5	0.9300
N1—C4	1.281 (3)	C6—C7	1.404 (3)
C1—C9'	1.489 (3)	C7—C8	1.360 (3)
C1—C2	1.502 (4)	C7—H7	0.9300
C1—H1A	0.9700	C8—C8'	1.392 (3)
C1—H1B	0.9700	C8—H8	0.9300
C2—C3	1.516 (4)	C8'—N9	1.380 (3)
C2—H2A	0.9700	C9—N9	1.457 (3)
C2—H2B	0.9700	C9—H9A	0.9599
C3—C4	1.495 (3)	C9—H9B	0.9599
C3—H3A	0.9700	C9—H9C	0.9599
C3—H3B	0.9700	N9—C9'	1.365 (3)
C4—C4'	1.449 (3)	C10—H10A	0.9599
C4'—C9'	1.377 (3)	C10—H10B	0.9599
C4'—C5'	1.443 (3)	C10—H10C	0.9599
			
N1—O1—H1X	97 (3)	C5'—C5—H5	120.6
C6—O2—C10	118.53 (19)	O2—C6—C5	124.8 (2)
C4—N1—O1	111.4 (2)	O2—C6—C7	114.6 (2)
C9'—C1—C2	109.3 (2)	C5—C6—C7	120.6 (2)
C9'—C1—H1A	109.8	C8—C7—C6	121.5 (2)
C2—C1—H1A	109.8	C8—C7—H7	119.3
C9'—C1—H1B	109.8	C6—C7—H7	119.3
C2—C1—H1B	109.8	C7—C8—C8'	118.3 (2)
H1A—C1—H1B	108.3	C7—C8—H8	120.9
C1—C2—C3	114.4 (3)	C8'—C8—H8	120.9
C1—C2—H2A	108.7	N9—C8'—C8	130.1 (2)
C3—C2—H2A	108.7	N9—C8'—C5'	108.78 (19)
C1—C2—H2B	108.7	C8—C8'—C5'	121.1 (2)
C3—C2—H2B	108.7	N9—C9—H9A	109.5
H2A—C2—H2B	107.6	N9—C9—H9B	109.5
C4—C3—C2	113.8 (2)	H9A—C9—H9B	109.5
C4—C3—H3A	108.8	N9—C9—H9C	109.5
C2—C3—H3A	108.8	H9A—C9—H9C	109.5
C4—C3—H3B	108.8	H9B—C9—H9C	109.5
C2—C3—H3B	108.8	C9'—N9—C8'	108.57 (17)
H3A—C3—H3B	107.7	C9'—N9—C9	126.4 (2)
N1—C4—C4'	118.3 (2)	C8'—N9—C9	125.0 (2)
N1—C4—C3	124.5 (2)	N9—C9'—C4'	109.8 (2)
C4'—C4—C3	117.2 (2)	N9—C9'—C1	125.1 (2)
C9'—C4'—C5'	107.03 (19)	C4'—C9'—C1	125.0 (2)
C9'—C4'—C4	120.8 (2)	O2—C10—H10A	109.5
C5'—C4'—C4	132.2 (2)	O2—C10—H10B	109.5
C5—C5'—C8'	119.57 (19)	H10A—C10—H10B	109.5
C5—C5'—C4'	134.6 (2)	O2—C10—H10C	109.5
C8'—C5'—C4'	105.8 (2)	H10A—C10—H10C	109.5
C6—C5—C5'	118.9 (2)	H10B—C10—H10C	109.5
C6—C5—H5	120.6		
			
C9'—C1—C2—C3	−46.4 (3)	C6—C7—C8—C8'	−0.6 (4)
C1—C2—C3—C4	50.2 (4)	C7—C8—C8'—N9	−178.2 (2)
O1—N1—C4—C4'	−179.6 (2)	C7—C8—C8'—C5'	0.2 (3)
O1—N1—C4—C3	−1.5 (4)	C5—C5'—C8'—N9	179.49 (18)
C2—C3—C4—N1	155.9 (3)	C4'—C5'—C8'—N9	−0.1 (2)
C2—C3—C4—C4'	−26.0 (3)	C5—C5'—C8'—C8	0.8 (3)
N1—C4—C4'—C9'	179.7 (2)	C4'—C5'—C8'—C8	−178.8 (2)
C3—C4—C4'—C9'	1.5 (3)	C8—C8'—N9—C9'	178.7 (2)
N1—C4—C4'—C5'	0.0 (4)	C5'—C8'—N9—C9'	0.2 (2)
C3—C4—C4'—C5'	−178.2 (2)	C8—C8'—N9—C9	−3.0 (4)
C9'—C4'—C5'—C5	−179.6 (2)	C5'—C8'—N9—C9	178.4 (2)
C4—C4'—C5'—C5	0.2 (4)	C8'—N9—C9'—C4'	−0.3 (2)
C9'—C4'—C5'—C8'	0.0 (2)	C9—N9—C9'—C4'	−178.4 (2)
C4—C4'—C5'—C8'	179.7 (2)	C8'—N9—C9'—C1	−179.9 (2)
C8'—C5'—C5—C6	−1.4 (3)	C9—N9—C9'—C1	1.9 (4)
C4'—C5'—C5—C6	178.1 (2)	C5'—C4'—C9'—N9	0.2 (2)
C10—O2—C6—C5	3.4 (4)	C4—C4'—C9'—N9	−179.64 (19)
C10—O2—C6—C7	−177.7 (2)	C5'—C4'—C9'—C1	179.9 (2)
C5'—C5—C6—O2	179.8 (2)	C4—C4'—C9'—C1	0.0 (3)
C5'—C5—C6—C7	1.0 (3)	C2—C1—C9'—N9	−157.9 (2)
O2—C6—C7—C8	−178.9 (2)	C2—C1—C9'—C4'	22.4 (3)
C5—C6—C7—C8	0.0 (4)		

### Hydrogen-bond geometry (Å, °)

**Table:**

*D*—H···*A*	*D*—H	H···*A*	*D*···*A*	*D*—H···*A*
C5—H5···N1	0.93	2.76	3.236 (3)	113
O1—H1X···O1^i^	0.827 (18)	2.54 (3)	3.177 (5)	134 (3)
O1—H1X···N1^i^	0.827 (18)	2.00 (2)	2.810 (4)	166 (4)

Symmetry codes: (i) −*x*+1, −*y*, −*z*.
